# Supplementary material for: A Lieb-like lattice in a covalent-organic framework and its Stoner ferromagnetism
Source: Nat Commun. 2019 May 17;10:2207. doi: 10.1038/s41467-019-10094-3 (PMC6525167; doi:10.1038/s41467-019-10094-3)
Supplement: Supplementary file 1 — Supplementary Information [file 41467_2019_10094_MOESM1_ESM.pdf]

Supplementary Information

A Lieb-Like Lattice in a Covalent-Organic Framework and Its Stoner Ferromagnetism

Supplementary Note 1

The eight bands around the Fermi level (five above and three below) without hole doping can be described by a eight-band TB model, using eight molecular orbitals on eight lattice sites. The corresponding effective Hamiltonian can be expressed as:

$$H = \begin{pmatrix} E_0+\Delta E_0 & t_0 V_{c\cdot ex} & t_0 V_{c\cdot ey} & 0 & 0 & 0 & 0 & 0 \\ t_0 V^*_{c\cdot ex} & E_0 & t'_0 V_{ex\cdot ey} & 0 & 0 & 0 & 0 & 0 \\ t_0 V^*_{c\cdot ey} & t'_0 V^*_{ex\cdot ey} & E_0 & 0 & 0 & 0 & 0 & 0 \\ 0 & 0 & 0 & E_1+\Delta E_1 & t_1 V_{c\cdot ex} & t_1 V^*_{c\cdot ex} & t_1 V_{c\cdot ey} & t_1 V^*_{c\cdot ey} \\ 0 & 0 & 0 & t_1 V^*_{c\cdot ex} & E_1 & t_1 V_{ex\cdot ex} & t'_1 V_{ex\cdot ey} & t'_1 V_{ey\cdot ex} \\ 0 & 0 & 0 & t_1 V_{c\cdot ex} & t_1 V^*_{ex\cdot ex} & E_1 & t'_1 V^*_{ey\cdot ex} & t'_1 V^*_{ex\cdot ey} \\ 0 & 0 & 0 & t_1 V^*_{c\cdot ey} & t'_1 V^*_{ex\cdot ey} & t'_1 V_{ey\cdot ex} & E_1 & t_1 V_{ey\cdot ey} \\ 0 & 0 & 0 & t_1 V_{c\cdot ey} & t'_1 V^*_{ey\cdot ex} & t'_1 V_{ex\cdot ey} & t_1 V^*_{ey\cdot ey} & E_1 \end{pmatrix}, \tag{1}$$

in which E_0 and E_0+ΔE_0 represent the on-site energy of the edge-center and corner sites for the three bands below the Fermi level, respectively; same for E_1 and E_1+ΔE_1 for the five bands above; t_0/t_1 and t'_0/t'_1 are the nearest-neighbor (NN) and the next NN hopping integrals, respectively; $V_{\alpha\cdot\beta}$ denotes the phase factor of the hopping between states of α and β, which can be the states at the corner (*c*), edge-center in x-axis (*ex*), or edge-center in y-axis (*ey*); the dimerization interaction δ between different states is included as a staggered hopping integral embedded in each $V_{\alpha\cdot\beta}$, e.g., $V_{c\cdot ex}$ is the phase factor of the hopping between the corner to the x-axis edge-center site, expressed as $\frac{t+\delta}{t}\mathrm{e}^{ik\cdot a_1}$ + $\frac{t-\delta}{t}\mathrm{e}^{-ik\cdot a_1}$, and $V_{c\cdot ey}$ is the phase factor of the hopping between the corner to the y-axis edge-center site, expressed as $\frac{t+\delta}{t}\mathrm{e}^{ik\cdot a_2}$ + $\frac{t-\delta}{t}\mathrm{e}^{-ik\cdot a_2}$, in which $a_{1,2}$ is the NN lattice vector.

Based on the energy diagram of the MOs calculated from Gaussian, we adjusted the $E_{0,1}$ and $E_{0,1}$+$\Delta E_{0,1}$, and then fit the TB band structures to the DFT results with different t_0/t_1, t'_0/t'_1, and δ values. We found $E_0 \approx$ -0.92 eV, $t \approx$ -0.13 eV, $t' \approx$ -0.01 eV, $\delta_0 \approx$ -0.04 eV, $\Delta E \approx$ 0.14 eV, $E_1 \approx$ 1.51 eV, $t_1 \approx$ -0.21 eV, $t'_1 \approx$ -0.01 eV, $\delta_1 \approx$ -0.03 eV, and $\Delta E_1 \approx$ 0.13 eV. It should be noted that these eight bands happened to be decomposed into two sets of bands, because

of the negligible interaction between them, as evidenced from DFT calculations. Specifically, DFT wavefunction analysis shows that these two sets of bands come from orbital basis of two sub lattices with little orbital overlap and large energy separation. Consequently, the 8-band TB Hamiltonian can be block diagonalized into two block of 3- and 5-band each (marked as red blocks in the figure below). It is for this reason that the 8-band TB model is effectively reduced to one 3-band and one 5-band model, which are further revealed to correspond to a Lieb-3 and a Lieb-5 band, respectively. The corresponding effective Hamiltonian can be expressed respectively as:

$$H_{Lieb-3} = \begin{pmatrix} E_0 + \Delta E & t \cdot V_{c-ex} & t \cdot V_{c-ey} \\ t \cdot V^*_{c-ex} & E_0 & t' \cdot V_{ex-ey} \\ t \cdot V^*_{c-ey} & t' \cdot V^*_{ex-ey} & E_0 \end{pmatrix}, \tag{2}$$

$$\text{and } H_{Lieb-5} = \begin{pmatrix} E_0 + \Delta E & t \cdot V_{c-ex} & t \cdot V^*_{c-ex} & t \cdot V_{c-ey} & t \cdot V^*_{c-ey} \\ t \cdot V^*_{c-ex} & E_0 & t \cdot V_{ex-ex} & t' \cdot V_{ex-ey} & t' \cdot V_{ey-ex} \\ t \cdot V_{c-ex} & t \cdot V^*_{ex-ex} & E_0 & t' \cdot V^*_{ey-ex} & t' \cdot V^*_{ex-ey} \\ t \cdot V^*_{c-ey} & t' \cdot V^*_{ex-ey} & t' \cdot V_{ey-ex} & E_0 & t \cdot V_{ey-ey} \\ t \cdot V_{c-ey} & t' \cdot V^*_{ey-ex} & t' \cdot V_{ex-ey} & t \cdot V^*_{ey-ey} & E_0 \end{pmatrix}, \tag{3}$$

Supplementary Note 2

To fit the DFT calculated band structure, we performed maximally localized Wannier functions (MLWF) fitting using the Wannier90 package. The projections were chosen as p_z states at the edge-center and corner sites. The MLWF fitted band structures are shown in Supplementary Figure 6, which agree perfectly with the DFT calculated results. The relaxed MLWFs states show the same results as the molecular informations calculated from DFT and Gaussian, which further confirm the Lieb-lattice nature of the Py(BCSB)$_2$ lattice. For the Lieb-3 lattice, the NN hopping term ($t \approx 0.11eV$) and the on-site energy difference ($\Delta E \approx 0.21eV$) are extracted from the fitted Hamiltonian file, which agrees very well with our tight-binding analyses. The

slight difference could potentially come from the consideration of higher-order interactions in Wannier fitting beyond the next NN interaction in our TB model.

Supplementary Note 3

Magnetic moments were introduced by doping the sp^2c-COF supercell with a doping concentration of (0.5h per unit cell) by changing the total number of electrons of the system while maintaining the charge neutrality with a compensating homogenous background charge. To deduce the Curie temperature of the sp^2c-COF, magnetic moments are treated as localized spins on pyrene unit. We first built a $1\times1\times2$ supercell to study the magnetic interaction between layers. Systems with both ferromagnetic (FM) and antiferromagnetic (AFM) spin configurations are calculated. After relaxation, both systems became FM state, indicating strong FM coupling between layers. Therefore, the Curie temperature is determined by the magnetic coupling strength within layers. Then, we construct a $\sqrt{2}\times\sqrt{2}\times1$ supercell and calculate the energy for both FM and AFM spin configurations (Supplementary Figure 9). Because the magnetic interactions between layers are always FM, we can just consider the magnetic interaction within layers by constructing a 2D square spin lattice formed by localized Py spins. The lattice Hamiltonian is defined as $H_s = -J_{ex}\sum_{i,j}\mathbf{S}_i \cdot \mathbf{S}_j$, where J_{ex} is the effective exchange coupling constant between Py units within the plane, $\mathbf{S}_{i,j}$ is the spin momenta of Py unit. Only nearest-neighbor (NN) exchange interactions were considered, the energy difference between different spin configurations (Supplementary Figure 9) can then be calculated by lattice summation, $\Delta H = H_{FM} - H_{AFM} = -8 * J_{ex}\mathbf{S}_i \cdot \mathbf{S}_j$. The exchange coupling (J_{ex}) among the Py ligands within the plane can be extracted by fitting the Heisenberg spin lattice model to the DFT-calculated energy difference between FM and AFM states (Supplementary Figure 9), which yield a value of $J_{ex} \approx$ 0.8meV. The positive J_{ex} indicate FM ground state, which gives rise to a Curie temperature of $\sim$ 9.3K. This is consistent with the experimental measured value

(~ 8.1K).

Supplementary Note 4

To study the preferred direction of the magnetic moments, we carried out non-collinear magnetic structure calculations as embedded in the VASP codes considering the spin-orbit coupling. Again, to induce localized magnetic moments, the bulk sp^2c-COF was doped with holes (0.5h per unit cell). Different magnetic moment directions were set by providing initial magnetic moments with x, y, and z components for each ion using the MAGMOM tag. The results are summarized in Supplementary Table 2, which show that the magnetic moments prefer to align in the direction perpendicular to the plane.

Supplementary Note 5

We also performed iodine doping calculations in comparison with experiments. Based on experimental results that the highest spin density is around 0.7 per unit cell, the hole doping concentration is expected to be in the range of [0 - 0.7] hole per unit cell. Therefore, we construct a $1\times1\times2$ supercell with one iodine ion to study the iodine doping effect. This structure is comparable with experiments as it has a doping concentration of 0.5 hole per unit cell and the interaction between iodine ions is properly avoided. We tested different adsorption sites (Supplementary Figure 10) and used the most stable one to study the magnetic properties of the system with the other two metastable structures examined (Supplementary Figure 11). All structural configurations show qualitative agreement with experiments, i.e., the generated magnetic moments are mainly localized on the corner pyrene ligands. We further tested the spin-orbit coupling effect of iodine, which has a negligible effect.

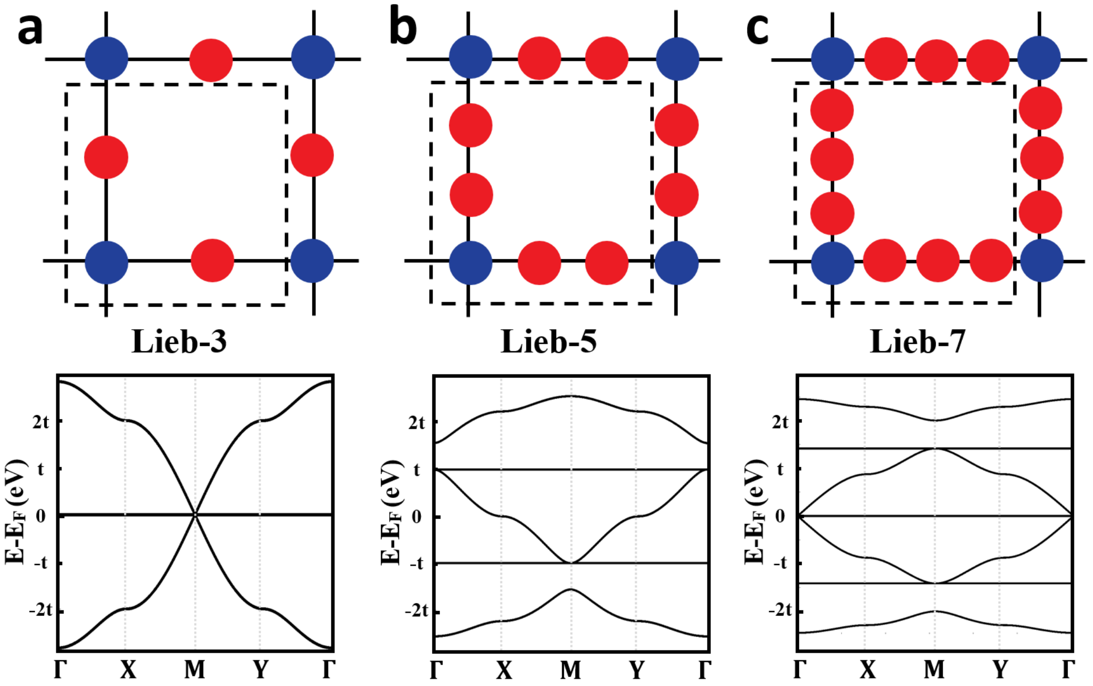

Supplementary Figure 1. Different ideal Lieb lattices. **a** Ideal Lieb-3 lattice (upper panel) with its corresponding electronic band structure along high-symmetry *k* paths (lower panel). **b** Same as (**a**) for a Lieb-5 lattice. **c** Same as **a** for a Lieb-7 lattice.

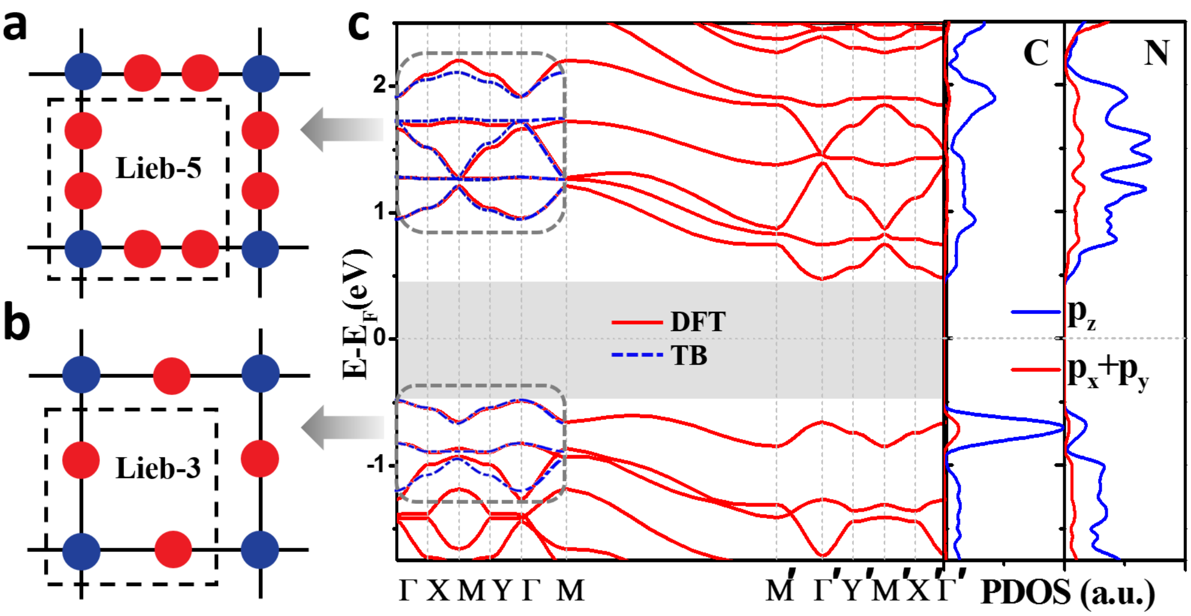

Supplementary Figure 2. Co-existence of the Lieb-3 and Lieb-5 bands in Py(BCSB)$_2$. a The Lieb-5 lattice. **b** The lieb-3 lattice. **c** Electronic band structure of the bulk Py(BCSB)$_2$ and the orbital-resolved projected density of states for C and N atoms. The red solid and blue dash bands represent the DFT calculated and TB fitted results, respectively. Capital letters on the *x*-axis with and without a prime indicate the high-symmetry *k*-points in the $k_z = 0$ and $k_z = 0.5$ planes, respectively.

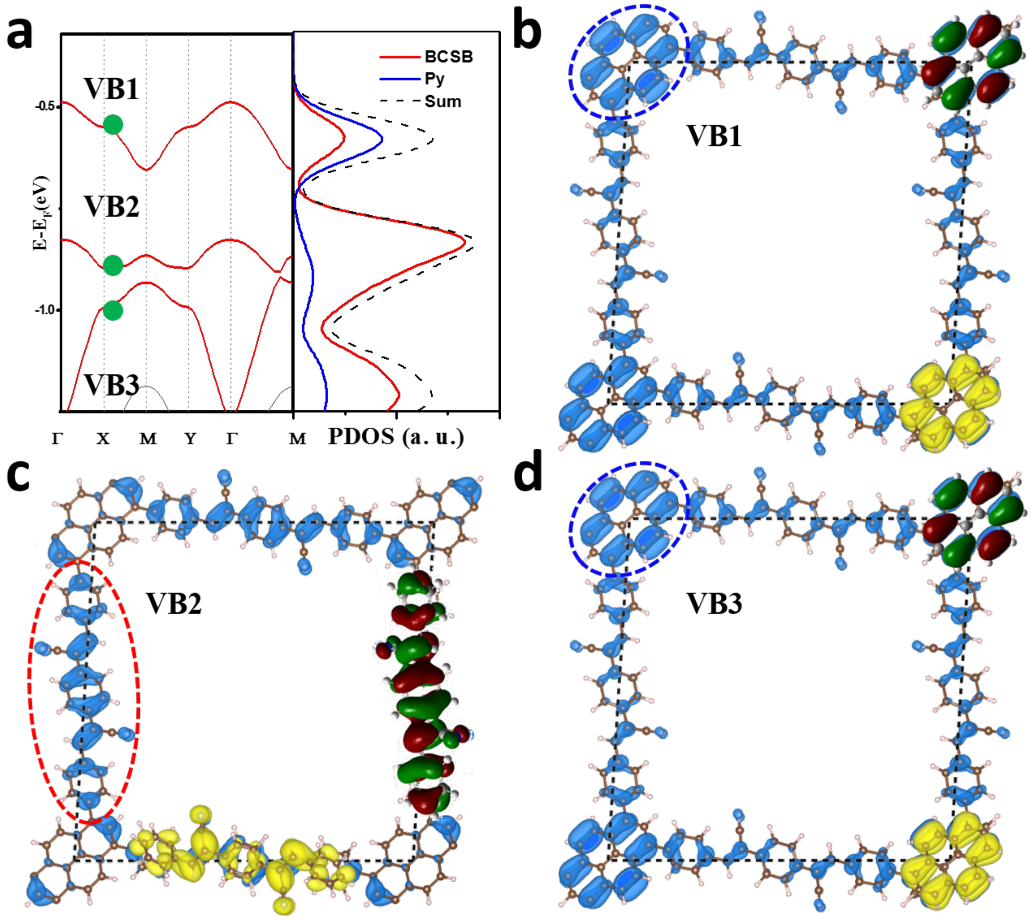

Supplementary Figure 3. Lieb-3 valence bands. a Enlarged Lieb-3 valence bands and projected local density of states for edge-center (BCSB) and corner (Py) sites below the Fermi level. **b**, **c**, and **d** are the band-resolved partial charge distribution near the *X* point for the three valence bands, i.e., VB1, VB2, and VB3 from DFT bulk calculation, respectively. The blue and red dashed ellipses highlight the localized charge states at the corner and edge-center sites, respectively. Gaussian and VASP calculated HOMOs are overlapped in **b**-**d** showing corresponding corner and edge-center states, respectively, that form the Lieb-3 lattice.

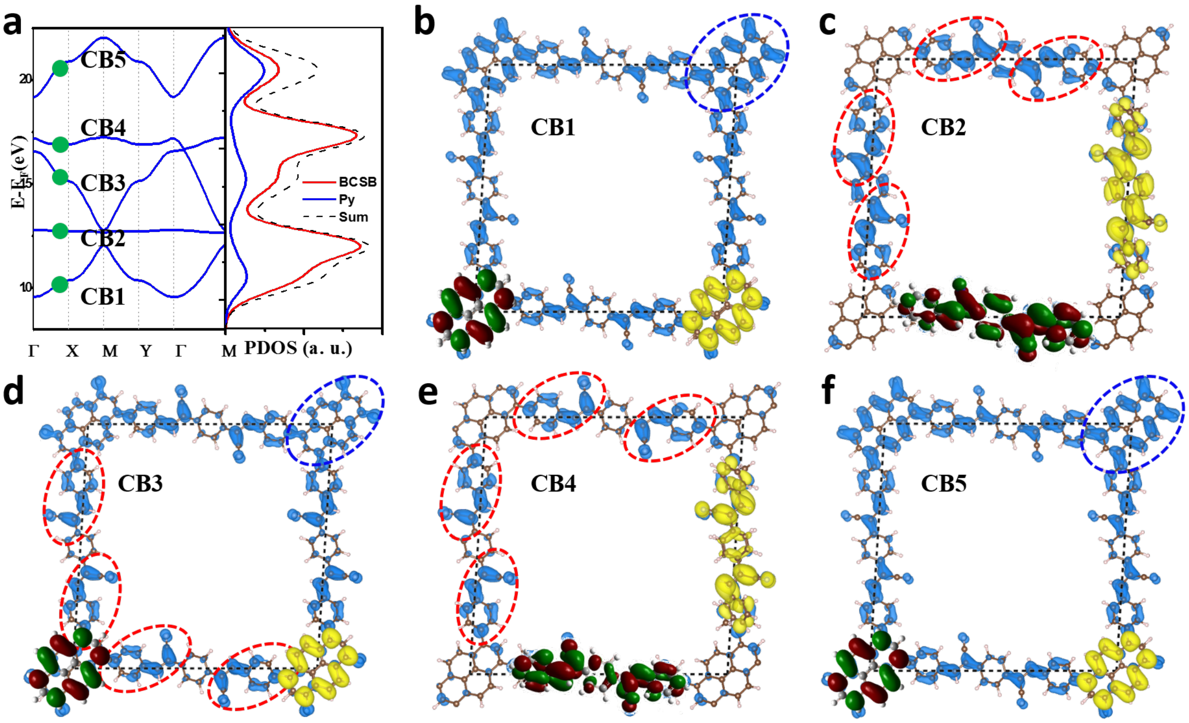

Supplementary Figure 4. Lieb-5 conduction bands. **a** Enlarged Lieb-5 conduction bands and projected local density of states for edge-center (BCSB) and corner (Py) sites above the Fermi level. **b**, **c**, **d**, **e**, and **f** are the band-resolved partial charge distribution near the *X* point for the five conduction bands, i.e., CB1, CB2, CB3, CB4, and CB5 from DFT bulk calculation, respectively. The blue and red ellipses highlight the charge localizations at the corner and edge-center sites, respectively. Gaussian and VASP calculated LUMOs are overlapped in **b**-**f** showing corresponding corner and edge-center states, respectively, that form the Lieb-5 lattice.

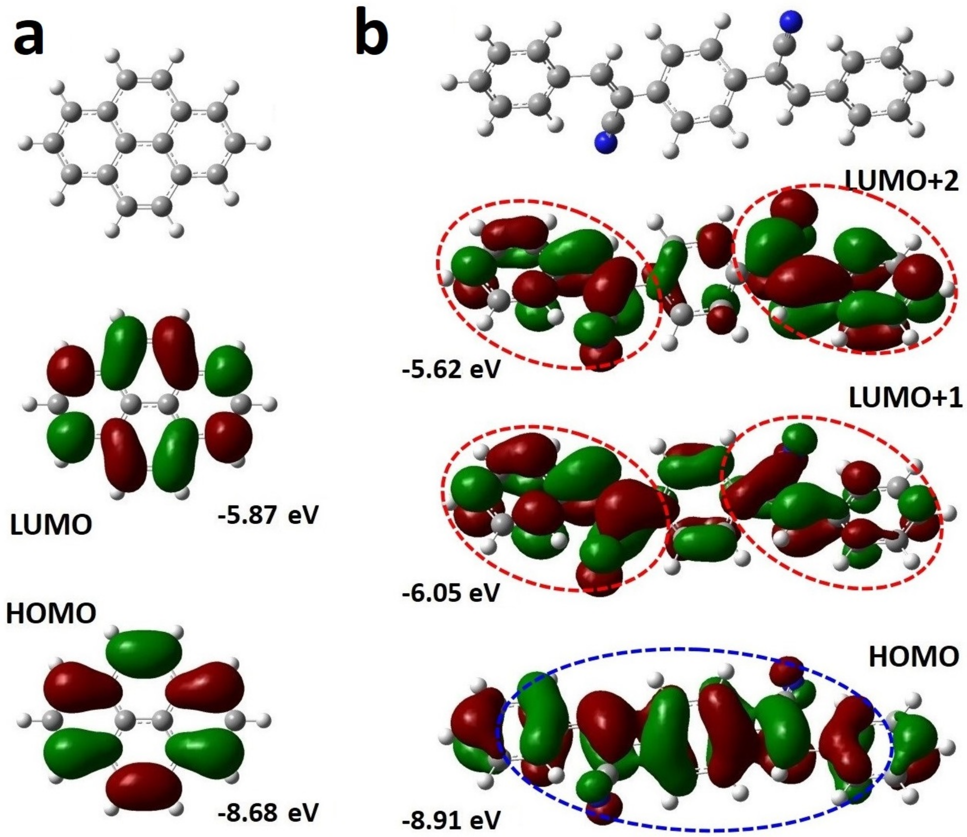

Supplementary Figure 5. Molecular information calculated using Gaussian codes. MO energy levels and the associated wavefunctions of **a** a Py and **b** a BCSB molecule. The red and blue ellipses highlight the charge localization of LUMOs and HOMO, respectively.

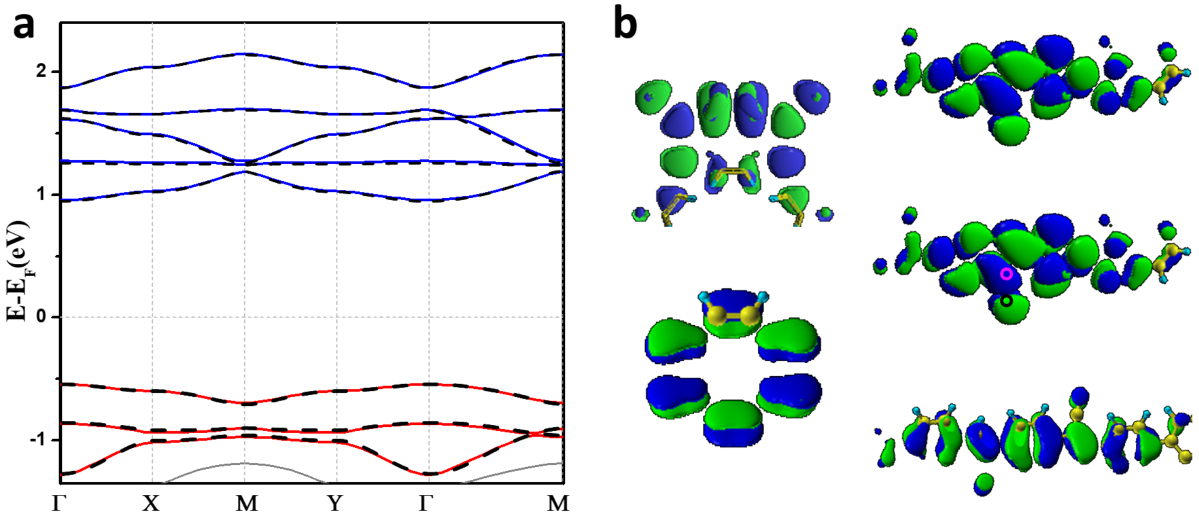

Supplementary Figure 6. Maximally localized Wannier functions (MLWF) fitting. a MLWF fitted band structure (black dashed lines) on topo of the DFT band structure (blue and red solid lines). **b** the MLWFs plot show great consistency with previous DFT calculations (Fig. S5).

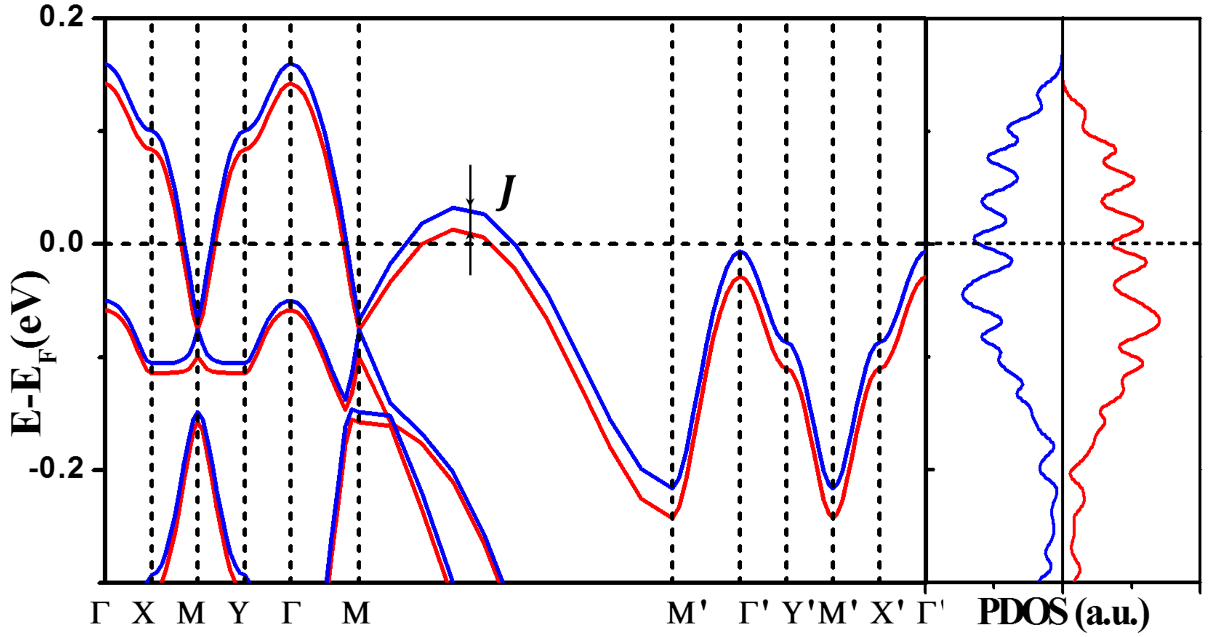

Supplementary Figure 7. Magnetism in hole-doped Py(BCSB)$_2$. Spin-polarized electronic band structure for k_z=0 and k_z = 0.5 planes and PDOS of the hole-doped Py(BCSB)$_2$.

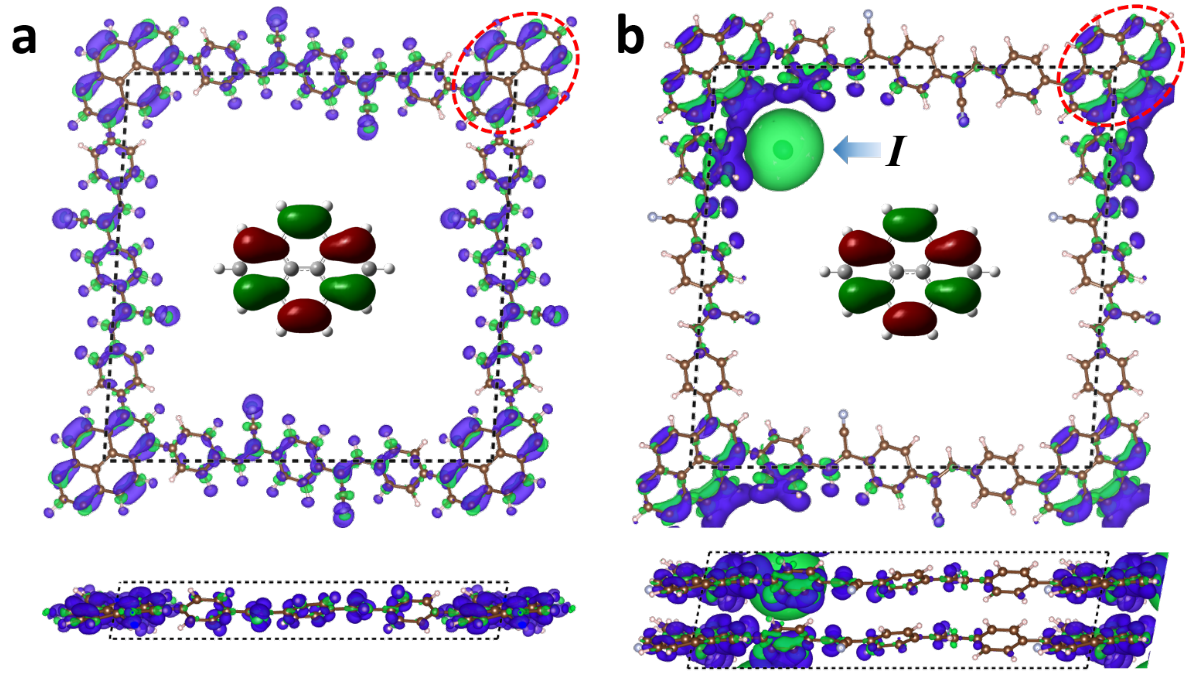

Supplementary Figure 8. Charge difference distribution. **a** The charge difference distribution of the monolayer Py(BCSB)$_2$ before and after the hole doping. **b** The charge difference distribution of the $1\times1\times2$ Py(BCSB)$_2$ supercell before and after the iodine (highlighted by blue arrow) doping. The purple and green color indicate the holes and the electrons, respectively. The red ellipses highlight the localization of the generated holes on the corner Pyrene ligands.

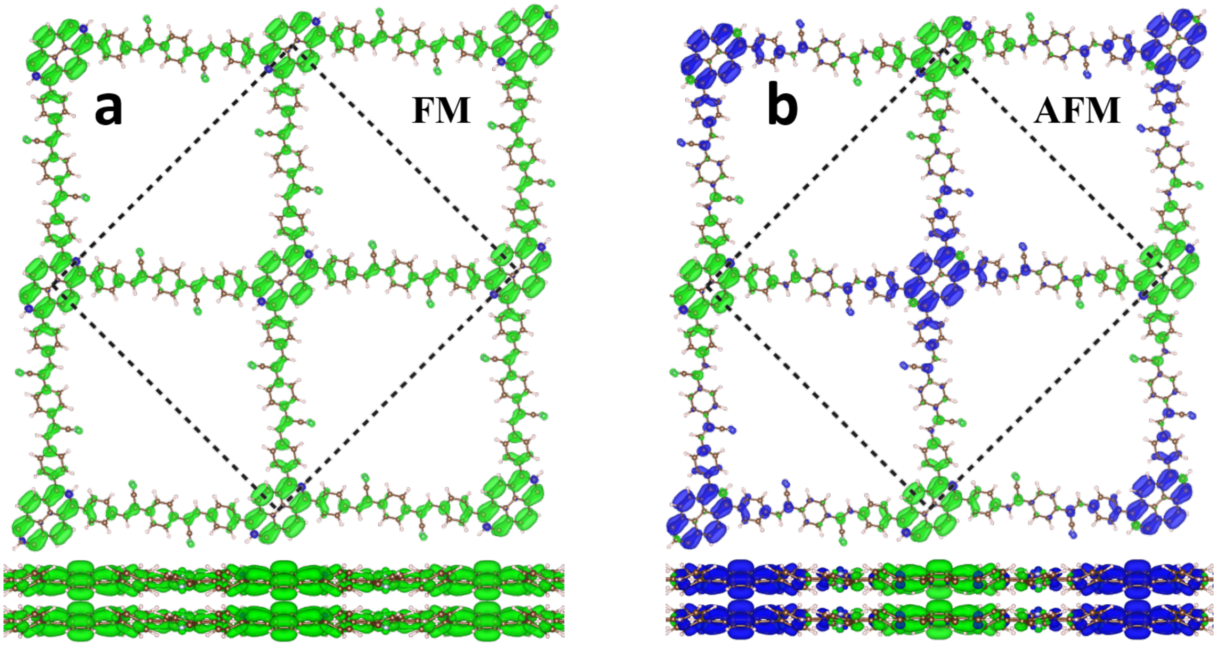

Supplementary Figure 9. Curie temperature approximation. a FM spin configuration and **b** AFM spin configuration of the $\sqrt{2}\times\sqrt{2}\times1$ Py(BCSB)$_2$ supercell with a doping concentration of one hole per supercell. The green and blue color denote different spin directions. The black dashed line indicates the supercell with two Py units.

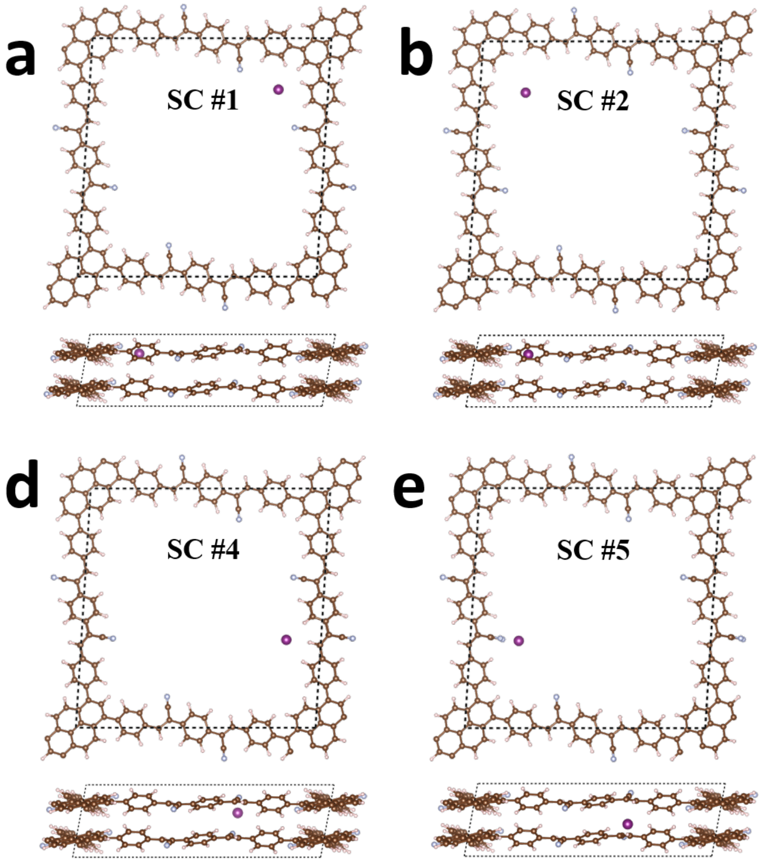

f

Structure Configurations	Energy difference ΔE E-E(most stable) eV
SC #1	**0.36**
SC #2	**0.00**
SC #3	**0.57**
SC #4	**0.04**
SC #5	**0.84**

Supplementary Figure 10. Structural configurations of iodine doping in 1×1×2 supercell. Structural configurations with iodine sitting at the **a** right and **b** left corner close to the Py ligands, **c** at the center of the supercell, at the **d** right and **e** left site close to the edge center. **f** The energy difference between difference structural configurations.

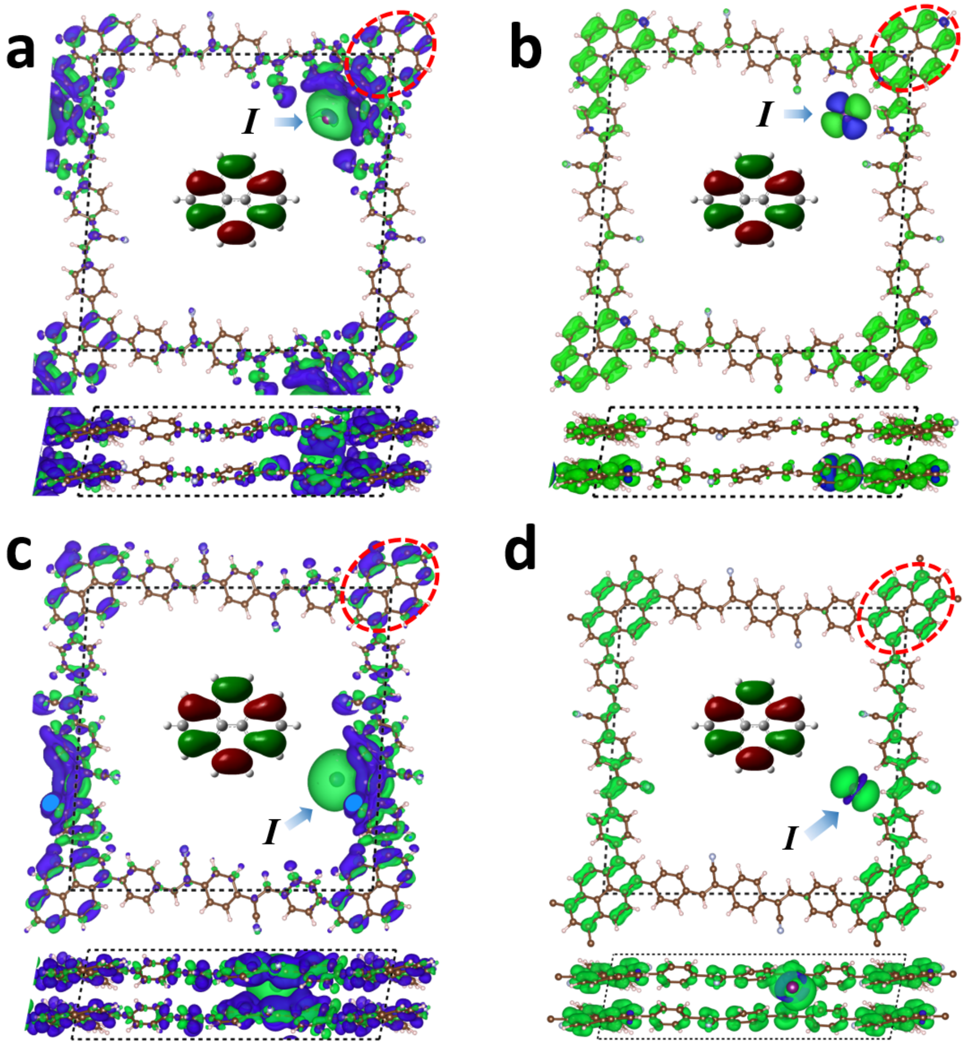

Supplementary Figure 11. Metastable structural configurations with iodine doping. a Top and side views of the charge difference distribution before and after the iodine (highlighted by blue arrow) doping for SC #1 with purple and green color representing the holes and electrons, respectively. **b** The spin distribution for SC #1. **c** and **d** The same as **a** and **b** for SC #4. The red ellipses highlight the localization of the generated holes as well as the induced spins on the corner Pyrene ligands.

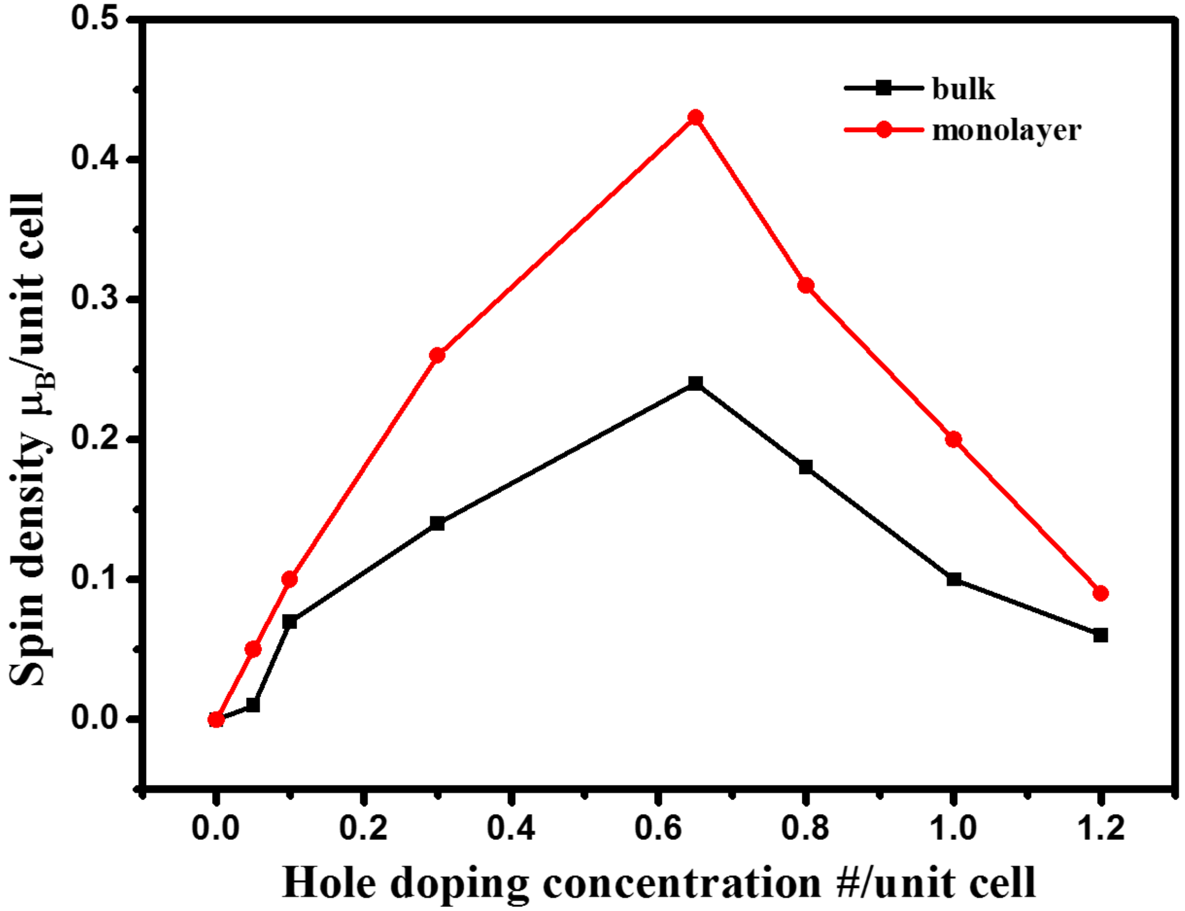

Supplementary Figure 12. Spin density as a function of hole doping concentration. The red and black curves represent the spin density variation with the hole doping concentration for the monolayer and the bulk $Py(BCSB)_2$, respectively.

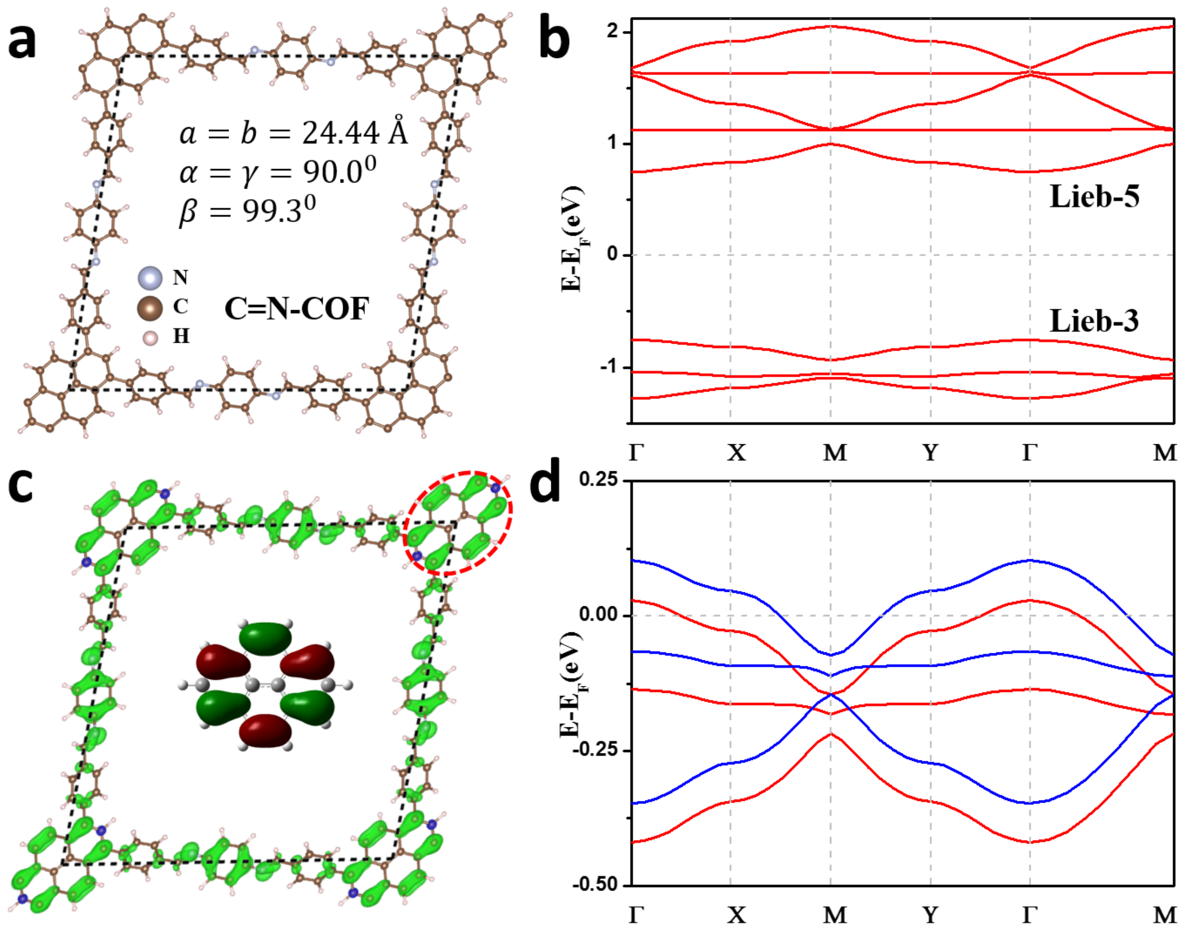

Supplementary Figure 13. Monolayer C=N-COF. a Monolayer structure of C=N-COF. **b** The electronic band structure along high-symmetry *k*-paths. **c** Spin distribution upon one hole doping. The red ellipse highlights the localization of the induced spins on the corner Pyrene ligands. **d** Spin-polarized valence band structure upon one hole doping.

Supplementary Table 1. Comparison of three key properties, i.e., bandwidth *W*, spin splitting *J*, and the highest magnetization *M*, for bulk $Py(BCSB)_2$, monolayer $Py(BCSB)_2$, and monolayer C=N-COF.

Property	*W* (meV)	*J* (meV)	*M* (μ_B/unit cell)
Bulk $Py(BCSB)_2$	362	20	0.23
Monolayer $Py(BCSB)_2$	183	65	0.43
Monolayer C=N-COF	178	80	0.64

Supplementary Table 2. Non-collinear magnetic structures calculations.

Magnetic moments directions (x, y, z)	Magnetic moment (x, y, z) μ_B	Total magnetic moment μ_B	Energy difference (meV)
(1, 0, 0)	(0.086, 0, 0)	0.086	0.1
(0, 1, 0)	(0, 0.085, 0)	0.085	0.1
(0, 0, 1)	(0, 0, 0.086)	0.086	0
(1, 1, 0)	(0.061, 0.061, 0)	0.086	0.2
(1, 1, 1)	(0.048, 0.049, 0.049)	0.085	0.2
